# Sexual dysfunctions in psoriatic patients

**DOI:** 10.3389/fpubh.2024.1339196

**Published:** 2024-04-17

**Authors:** Julia Nowowiejska, Agata Karny, Miłosz Nesterowicz, Piotr Purpurowicz, Anna Baran, Tomasz W. Kaminski, Zbigniew Purpurowicz, Iwona Flisiak

**Affiliations:** ^1^Department of Dermatology and Venereology, Medical University of Białystok, Białystok, Poland; ^2^Department of Urology and Urological Oncology, Municipal Polyclinical Hospital, Olsztyn, Poland; ^3^Pittsburgh Heart, Lung and Blood Vascular Medicine Institute, University of Pittsburgh, Pittsburgh, PA, United States

**Keywords:** psoriasis, sexual dysfunction, FSFI, IIEF, female sexual function index, international index of erectile function

## Abstract

**Introduction:**

Psoriasis is one the most common skin diseases associated with a great decrease in the quality of patients’ lives.

**Methods:**

We aimed to study sexual dysfunctions in psoriatic patients using the Female Sexual Function Index (FSFI) for women and the International Index of Erectile Function (IIEF) for men via an anonymous online survey. The study included 80 psoriatic patients and 75 controls without dermatoses.

**Results:**

There was a downward trend in the total IIEF score in psoriatic men compared to controls. 58% of male patients and 76% of controls had a normal IIEF score. There was no significant difference in IIEF between patients treated and not with systemic agents. 62% of female patients had a decreased FSFI score, whereas in the control group, the majority of subjects (54%) had a normal FSFI score. There was no significant difference in FSFI score between patients and controls. Female patients treated with systemic antipsoriatic agents had significantly worse lubrication, satisfaction with sexual life, and pain.

**Discussion:**

Our study has shown that the majority of questioned female psoriatic patients had sexual dysfunction according to FSFI, particularly they had worse satisfaction with sexual life and less sexual desire compared to women without psoriasis. The majority of male patients did not have sexual dysfunction according to IIEF, however, they had significantly worse overall satisfaction with sexual life and confidence to keep an erection. Systemic antipsoriatic treatment does not probably influence sexual dysfunctions in men but it does in women although we were not able to assess the severity or resolution of lesions after those treatments. However embarrassing, psoriatic patients should be questioned about their sexual lives by dermatologists, and more studies are needed to explore this matter.

## 1 Introduction

Psoriasis is a chronic inflammatory dermatosis of uncertain cause classified as a systemic ailment based on the most recent research. It is prevalent in the global adult population at about 2% (1). This dermatosis frequently co-occurs with metabolic syndrome and other serious disorders. Due to chronic inflammation, immune disorders, genetic factors, and a variety of biologically active substances secreted by adipose tissue, psoriatic patients are at higher risk of obesity, diabetes mellitus, hypertension, dyslipidemia, and atherosclerosis (2). Furthermore, these illnesses increase the probability of cardiovascular events (1). Additionally, previous research shows that the patients suffer from insomnia and have poor sleep quality (3). The comorbidities mentioned earlier enhance directly the risk of sexual dysfunction in both male and female patients (4). Moreover, cigarette smoking, excessive alcohol consumption, anxiety, and depression were also found to be significantly more common in individuals with psoriasis. Dermatological patients in particular are known to be vulnerable to mental discomfort, largely because skin lesions are easily noticeable and attract people’s attention. The fact that this illness is often long-lasting adds to the emotional stress. Due to the reasons presented above and the psychological side of the illness, it becomes clear that psoriasis is associated with the occurrence of sexual dysfunctions.

The World Health Organization (WHO) states that sexual health does not only mean the absence of disease or dysfunction but it is demonstrated by physical, emotional, mental, and social well-being concerning sexuality (5). Unfortunately, this is still a topic that is not frequently discussed.

The physiology of human sexuality depends on the cooperation of the neurotransmitters that either stimulate (e.g., dopamine, adrenaline,) or inhibit (serotonin endogenous opioids) the sexual response, hormones (e.g., testosterone, adrenal androgens, estrogens, progesterone,), as well as the functioning of the cardiovascular and nervous systems. Furthermore, relationships, social and cultural situations, and medications are additional factors influencing sexual satisfaction and health. However, hypoactive sexual desire disorder, sexual arousal dysfunction, orgasmic dysfunction, relaxation phase, as well as genital-pelvic pain dysfunction that can appear may lead to reduced quality of life (6–8).

About 45% of female patients in the general population report having problems in the sexual sphere. These difficulties are psychological as well as hormonal and general health-related. Among dysfunctions in men, erectile dysfunction is common, it can be organic (neurogenic, vascular, hormonal, or drug-related) or of a psychological nature (7, 8).

It is important to emphasize that psoriasis frequently has an impact on patient’s appearance, making it challenging for them to interact socially and build relationships (9). However, in individuals with the disease, the development of sexual dysfunction may be dependent on the location of the skin lesions, the severity of the condition, the adverse effects of medications, the existence of comorbidities, emotional factors, and psychiatric conditions (10, 11).

It is presumed that in female psoriatic patients, it is the desire that is an aspect of intimate life most affected (12). It is thought that skin lesions may not always have a direct effect on the appearance of dysfunction, but may also cause patients to feel less physically attractive. The occurrence of genital skin lesions and physical discomfort can harm a woman’s sexual functioning (10, 13). Symptoms such as burning and pruritus can negatively affect sexual activity in these patients (12, 14, 15). The severity of the condition, may also affect sexual dysfunctions (16). Research has shown that individuals with psoriasis have a lower quality of sexual life and a significantly lower acceptance of their physical appearance (12).

Psoriasis can lead to erectile dysfunction in male patients, the incidence of which increases with the duration of the condition. However, comorbidities such as anxiety and depressive disorders, metabolic syndrome, and smoking are likely to be the direct cause of this dysfunction (12, 17). Reduced total testosterone and increased estradiol in the blood are also reported in male patients (18).

Topical and systemic antipsoriatic treatment is considered to be another cause of sexual dysfunctions (12, 19). For example, methotrexate can lead to erectile dysfunction and reduced libido (20, 21). Medications taken due to psoriasis comorbidities [e.g., antidepressants, beta-blockers, thiazide diuretics, are also highlighted as potential causes of sexual dysfunctions (12)]. Nevertheless, intensive general treatment of psoriasis, including biologic therapy, which significantly reduces the severity of the disease, results in an improvement in the sexual function of patients (22–24).

We aimed to evaluate sexual dysfunction among patients with psoriasis who are one of the most commonly appointed by dermatologists both in the hospital, as well as in ambulatory care. More in-depth knowledge about the scale of such dysfunction in this specific group of patients would enable their better management.

## 2 Materials and methods

The study included 80 psoriatic patients (33 men and 47 women) and 75 controls (21 men and 54 women) without skin diseases. The exclusion criteria were: age under 18 years old, pregnancy, breastfeeding, history of urological or gynaecological procedures (prostatectomy, cystectomy, transurethral resection of the prostate – TURP, sling procedures, bladder neck suspension, hysterectomy, ovariectomy; moreover, patients with any surgical procedures in the anogenital area within 6 weeks after the procedure were excluded), intake of medications affecting sexual functions (beta-adrenolytics, thiazide diuretics, spironolactone, digoxin, opiates, antiepileptics, selective serotonin reuptake inhibitors, serotonin-norepinephrine reuptake inhibitors, benzodiazepines, 5α-reductase inhibitors), depression, alcohol abuse and smoking (more than 20 cigarettes per day). All patients received topical antipsoriatic treatment and 30 patients additionally received systemic antipsoriatic agents (methotrexate, acitretin, cyclosporin A, or biological agents).

### 2.1 Data collection

The study was conducted as an anonymous online survey among patients with the diagnosis of psoriasis made by a dermatologist (study group) and subjects free from any skin disease (control group, established after the collection of the study group, no later than 1 month after the study group data was generated to exclude any bias potentially associated with the season of the year). The survey was designed this way so that more people are willing to take part in the study, despite the embarrassment associated with the delicate nature of the investigated matter.

The survey contained demographic questions, questions regarding patients’ medical history, including drug intake and gynaecological or urological diseases/procedures, Female Sexual Function Index (FSFI) for women, International Index of Erectile Function (IIEF) for men, basic questions regarding psoriasis and Dermatology Life Quality Index (DLQI) for patients.

FSFI consists of 19 questions which are divided into 6 domains: desire, arousal, lubrication, orgasm, sexual satisfaction, and pain. Questions are awarded 0–5 points, the higher the score, the less dysfunction. The minimal score is 2, the maximal score is 36. IIEF consists of 15 questions which are divided into 5 domains: erectile function, intercourse satisfaction, orgasmic function, sexual desire, and overall satisfaction. Questions are awarded 0–5 points, the higher the score, the less dysfunction. The minimal score is 1 and the maximal is 30.

We divided patients and controls depending on the normal or abnormal total score in FSFI/IIEF. The cut-off was ≦26 points for FSFI and ≦21 points for IIEF.

We chose one question from each questionnaire summarizing the sexual life quality: how satisfied have you been with your overall sexual life? – question no 16 from FSFI and no 13 from IIEF. We assigned points from 1 to 5 for each answer, the higher the number, the better satisfaction. The whole list of questions from the questionnaires is presented in the Supplementary files.

Patients have also been given Alcohol Use Disorder Identification Test (AUDIT) to detect alcohol abuse and questioned about smoking habits, as alcoholism and smoking more than 20 cigarettes per day have been identified as risk factors for sexual dysfunctions (12, 25).

### 2.2 Statistical analysis

The data underwent cross-sectional statistical analysis. The Shapiro–Wilk test was employed to assess normality, with subsequent processing diverging based on the distribution characteristics. Specifically, data conforming to a normal distribution underwent analysis through the Student t-test, whereas non-Gaussian data were subjected to Mann–Whitney non-parametric analysis. Binary data, on the other hand, were assessed through chi-square testing. For comparisons among multiple sub-groups, one-way analysis of variance (ANOVA) was utilized. The correlations between variables were determined using Spearman’s rank correlation. Unless otherwise specified, the data is presented as mean ± SEM, with statistical significance defined as a two-tailed *p*-value of <0.05. All computations were executed using GraphPad 9 Prism Software (GraphPad Software, San Diego, CA, United States), while the power of the analysis was estimated with StatMate Software (GraphPad Software, San Diego, CA, United States). Additionally, all the graphs were generated using GraphPad 9 Prism software.

## 3 Results

The basic characteristics of patients and controls are presented in Table 1.

**Table 1:** Basic characteristics of patients and controls.

Parameter	Controls (*n* = 75)	Study Group (*n* = 80)
Sex (M/F)	21/54	33/47 (NS)
Age (years)	29.87 ± 2.05	34.29 ± 1.87*
Sexual orientation (non-heterosexual/heterosexual)	3/72	2/78 (NS)
Partnership (Y/N)	45/30	55/25 (NS)
Anogenital skin lesions (Y/N)	N/A	39/41
Concomitant psoriatic arthritis (Y/N)	N/A	13/67
DLQI	N/A	11.96 ± 0.96

NS, non-significant; Y, yes; N, no; N/A, not applicable; n, number of subjects in the group. * means statistically significant difference with *p* < 0.05.

There was no statistically significant difference between patients and controls in terms of gender, sexual orientation, and partnership status (NS). There was a slightly significant difference in terms of age between patients and controls (*p* < 0.05) which was due to the difficulties in obtaining information from younger psoriatic patients. Almost 49% of patients presented psoriatic skin lesions in the anogenital area. Only 16.25% of all patients had concomitant psoriatic arthritis (PsA). The majority of patients lived in towns and cities (78.75%), including 15% in cities with more than 500,000 inhabitants. The rest of the patients lived in the countryside (21.25%).

The mean DLQI was 11.96 ± 0.96 which means a very large effect on the quality of life. Considering the particular question (no 9) – how did skin lesions affect your sexual life? – 22.5% of patients responded that very much, 10% of patients – a lot, and 17.5% of patients – a little. There was a strong correlation between question no 9 and the presence of lesions in the anogenital area (*R* = 0.425; *p* < 0.0001).

We did not find any significant correlation between FSFI or IIEF scores and the diagnosis of psoriatic arthritis.

### 3.1 Men

Men have undergone evaluation using the IIEF questionnaire. Supplementary Table S1 presents the summary of IIEF scores.

Patients obtained significantly lower results in questions 13, 14, and in the overall satisfaction score (*p* < 0.05), meaning significantly less satisfaction. Moreover, they had significantly lower scores in question no 15 meaning they had significantly less confidence that they could keep the erection. As for the total score of IIEF, there was a downward trend in patients observed (*p* = 0.1).

**Figure 1:**
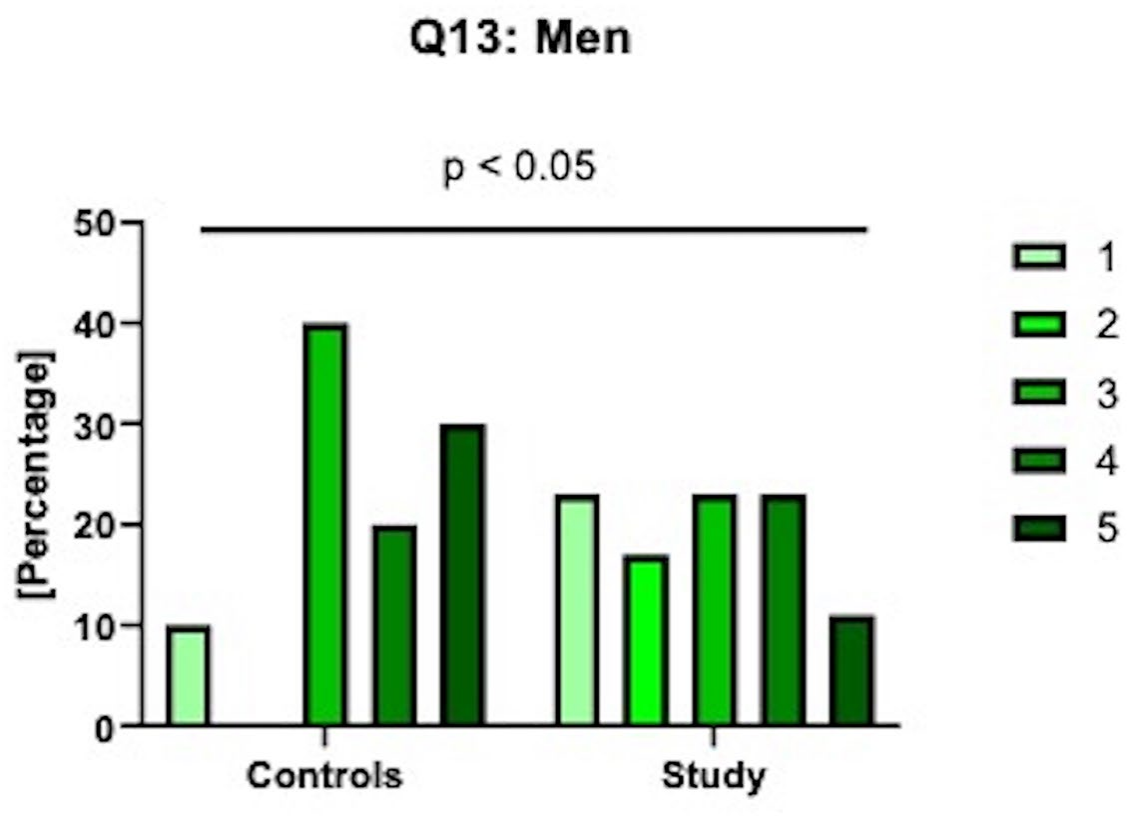
Specific results of the 13th question in IIEF: how satisfied have you been with your overall sexual life? 1 means very dissatisfied; 2 - moderately dissatisfied, 3 - equally satisfied & dissatisfied; 4 - moderately satisfied; 5 - very satisfied.

As for the specific results of question no 13 – how satisfied have you been with your overall sexual life? – there was a significant difference between the answers in all the groups (*p* < 0.05). The patients had a significantly higher proportion of answers awarded 1 point meaning worse satisfaction with sexual life (Figure 1).

**Figure 2:**
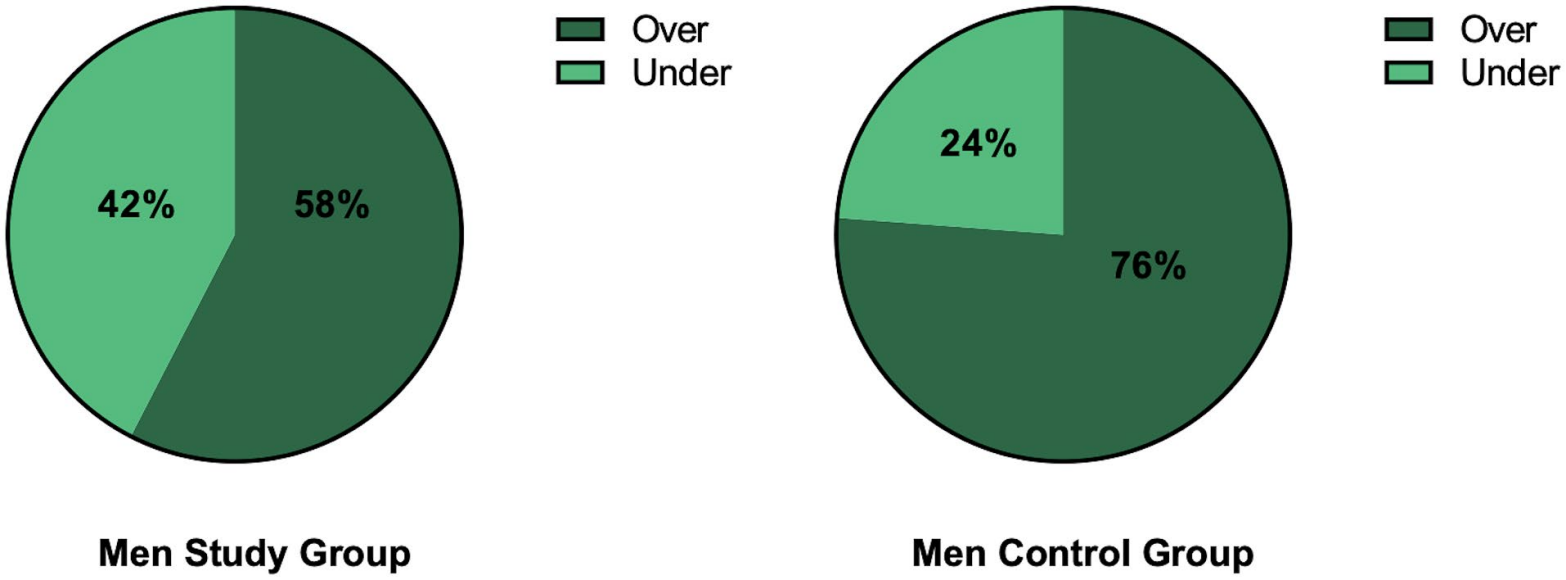
The division of patients and controls into two groups: with or without sexual dysfunctions based on IIEF with the cut-off ≦21 points. Over means the score higher than 21 points, under means equal to or less than 21.

Analyzing the cut-off value in IIEF, the patients and controls were divided into two groups –≦21 points and over 21 points– indicating sexual dysfunctions or their lack, respectively (Figure 2).

The majority of patients (58%) and controls (76%) had a normal score in IIEF meaning no sexual dysfunction. However, the proportion of subjects with normal scores was higher among the control group.

Patients were also divided into two groups according to the kind of antipsoriatic treatment they received – systemic vs. only topical (Supplementary Table S2).

There was no significant difference in male patients in the results of the IIEF questionnaire depending on the type of antipsoriatic therapy (NS).

We noticed a negative correlation between psoriasis duration and erectile function (*R* = −0.348, *p* < 0.05), confidence to keep the erection (Q15; *R* = −0.353, *p* < 0.05), and the number of attempts to sexual intercourse (Q6; *R* = −0.334, *p* < 0.05).

After the analysis of particular questions from the DLQI questionnaire, we found a positive correlation between Q9 and erectile function (*R* = 0.459, *p* < 0.0001).

There was no correlation between IIEF and the presence of lesions in the anogenital area (NS).

### 3.2 Women

Women have undergone evaluation using the FSFI questionnaire. Supplementary Table S3 presents the summary of FSFI scores.

Female patients obtained significantly lower results in question 2 (*p* < 0.05) and a downward trend concerning the desire domain in total (*p* = 0.052). There was a downward trend in patients observed about question 3 regarding arousal (*p* = 0.068). The patients also had significantly lower scores in question no 16 regarding the satisfaction with sexual life (*p* < 0.05).

**Figure 3:**
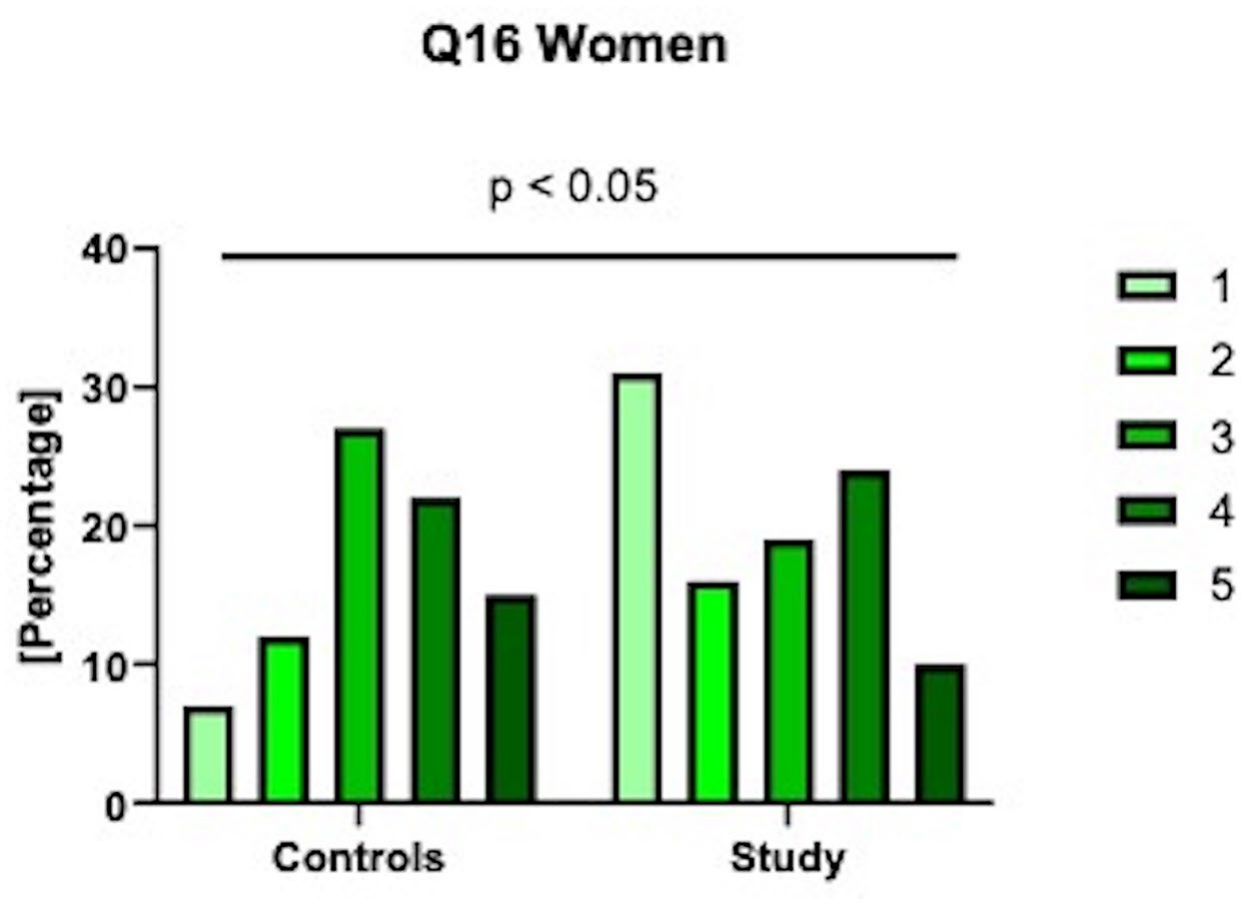
Specific results of the 16^th^ question in FSFI: how satisfied have you been with your overall sexual life? 1 means very dissatisfied; 2 - moderately dissatisfied, 3 - equally satisfied & dissatisfied; 4 - moderately satisfied; 5 - very satisfied.

As for the specific results of question no 16 – how satisfied have you been with your overall sexual life? – there was a significant difference between the answers in all the groups (*p* < 0.05). The patients had a significantly higher proportion of answers awarded 1 point meaning worse satisfaction with sexual life (Figure 3).

**Figure 4:**
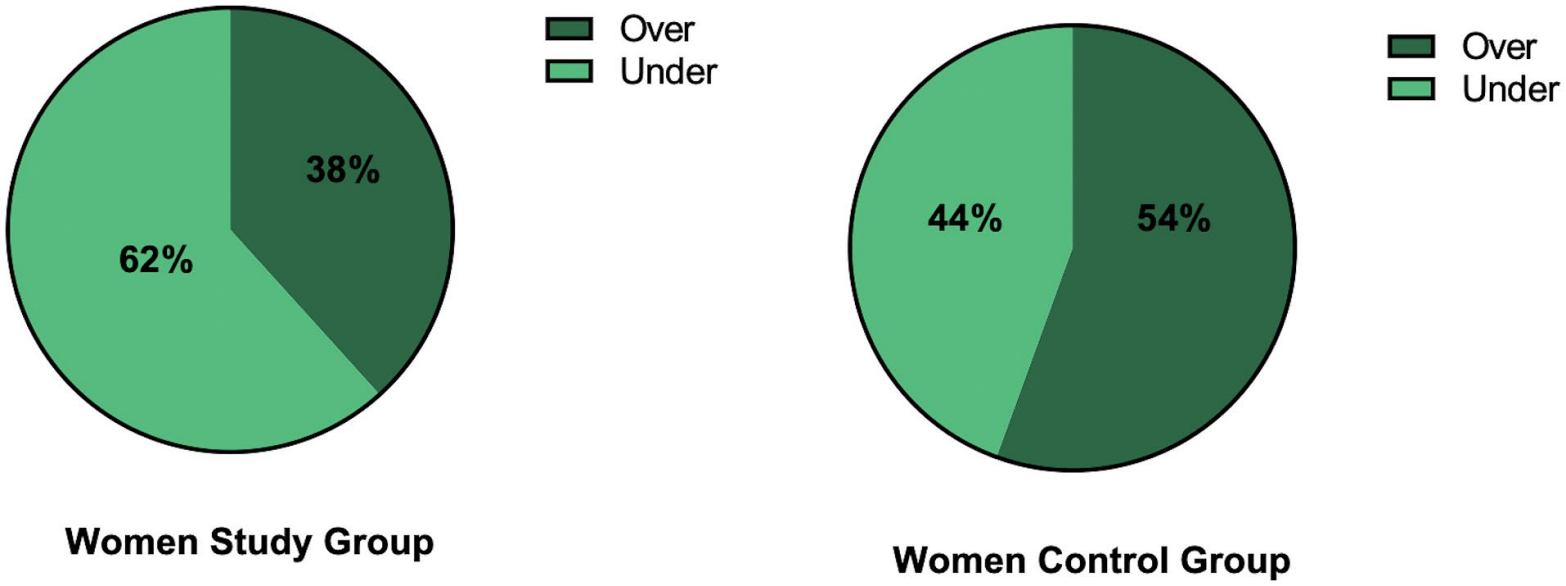
The division of patients and controls into two groups: with or without sexual dysfunctions based on FSFI with the cut-off of ≦26 points. Over means the score higher than 26 points, under means equal to or less than 26.

Analyzing the cut-off value in FSFI, the patients and controls were divided into two groups – ≦26 points or over 26 points – indicating sexual dysfunctions or their lack, respectively (Figure 4).

The majority of patients (62%) had a decreased score in FSFI meaning the existence of sexual dysfunction. In the control group, the majority of subjects (54%) had a normal FSFI score.

Patients were divided into two groups according to the kind of antipsoriatic treatment they received – systemic vs. topical (Supplementary Table S4).

Female patients undergoing systemic treatment had significantly lower scores in questions 7, 9, 10, and a total score in the lubrication domain (*p* < 0.05). Moreover, they had significantly lower scores in questions 15, 16, and total scores in the satisfaction domain (*p* < 0.05), as well as in the pain section (*p* < 0.05). We obtained a downward trend for the correlation between the lubrication and acitretin intake (*p* = 0.079).

We did not observe any significant correlations between the psoriasis duration and FSFI scores (NS). There was also no correlation between FSFI and the presence of lesions in the anogenital area (NS).

We separately analyzed all questions from the DLQI questionnaire and found several significant correlations. Question no 1 – how itchy, sore, painful, or stinging has your skin been? – was negatively correlated with the desire domain score from FSFI (*R* = −0.41; *p* < 0.01); the orgasm domain (*R* = −0.34; *p* < 0.05); and the total FSFI score (*R* = −0.314; *p* < 0.05). Question no 2 – how embarrassed or self-conscious have you been because of your skin? – was negatively correlated with the desire domain (*R* = −0.34; *p* < 0.05); orgasm domain (*R* = −0.41; *p* < 0.01); and total FSFI score (*R* = −0.38; *p* < 0.01). Question no 8 – how much has your skin created problems with your partner or any of your close friends or relatives? – was negatively correlated with the desire domain (*R* = −0.46; *p* < 0.01); arousal domain (*R* = −0.31; *p* < 0.05); and total FSFI score (*R* = −0.33; *p* < 0.05). Question no 9 – how much has your skin caused any sexual difficulties? – was negatively correlated with the desire domain (*R* = −0.38; *p* < 0.01); arousal domain (*R* = −0.36; *p* < 0.05); lubrication domain (*R* = −0.31; *p* < 0.05); orgasm domain (*R* = −0.36; *p* < 0.05); pain domain (*R* = −0.39; *p* < 0.01); and total FSFI score (*R* = −0.4; *p* < 0.01).

## 4 Discussion

However, much is said about the decreased quality of psoriatic patients’ lives, not as much attention is paid to the sexual lives of such patients. The main research topics in terms of psoriasis comorbidity are metabolic syndrome, psoriatic arthritis, or inflammatory bowel disease (26). Sexual dysfunctions are less studied. Partially, it may be due to the embarrassing nature of these problems which makes patients not willing to take part in such research. This is why we chose an anonymous online survey. As far as we are concerned, this is the third study on sexual dysfunctions in our country, however the first to use the control group for comparison and with the biggest sample size.

So far there have been several studies that aimed at assessment of sexual dysfunctions prevalence and severity in psoriatic patients. According to available analyses originating from different countries, the prevalence of sexual dysfunctions in psoriatics in general (both in women and men) is reported in a very wide range between 22.6–71.3% (16), whereas only in psoriatic women is estimated to be between 48–68%, and only in men – between 21–61.5% (27, 28). One systematic review that took into account 15 separate studies that included from 24 to 12,300 patients, revealed that in all of them, there was a higher frequency of sexual dysfunctions among psoriatics (16).

Even despite the above-mentioned evidence, there are no guidelines on the screening or management of sexual dysfunctions in psoriatic patients, neither international nor local. Probably the most common and easy-accessible tool is DLQI which includes one question regarding the influence of psoriasis on sexual life. One study reported that at least 43% of psoriatics claim that their doctor does not show any interest in the matter of sexual dysfunctions due to the dermatosis (29) and in another study, only 9% of psoriatic stated that they have been indeed asked about such problems (30).

In our study, a significant number of all patients – 50% - responded that psoriasis affects their sexual life in the DLQI questionnaire. There was a significant correlation between dermatological quality of life and the presence of psoriatic lesions in the anogenital region. Similar observations were made by Meewuis et al. (29).

The majority of male psoriatic patients (58%) had a normal score in IIEF according to the rules of the questionnaire however the rate of normal scores was lower compared to the controls (76%). At the same time, there was a downward trend in male patients’ scores in IIEF compared to controls. That would indicate that however there is no hard evidence for sexual dysfunctions in male psoriatic patients in our study, psoriatic patients are more prone to such problems than subjects without dermatoses. Other teams, such as Türel Ermertcan et al. observed significantly lower total scores of IIEF in psoriatic patients (31). In a big meta-analysis of erectile dysfunction in psoriatics by Wu et al., it was confirmed that psoriasis is indeed associated with an elevated risk of such dysfunctions (32).

The most compromised domain in male patients from our cohort compared to controls was overall satisfaction with sexual intercourse, similar to Türel Ermertcan et al. (31). Moreover, they had significantly lower confidence that they could keep the erection (question 15) compared to subjects without skin diseases.

The majority of female psoriatic patients (62%) had abnormal – decreased – total score in FSFI indicating the presence of sexual dysfunction. At the same time, there was no significant difference between patients and controls in terms of FSFI total score. Female patients had significantly lower sexual desire (question 2) and satisfaction with their sexual life (question 16) than controls. Similar findings were reported by Nguyen et al., who found additionally significantly worsened arousal and pain (33), whereas Türel Ermertcan et al. – arousal and orgasm (31). Kuzriki et al. also pointed to decreased desire as the one that is mainly affected (34). Meeuwis and Türel Ermertcan et al. obtained similar results to ours with decreased total FSFI in psoriatic women (29, 31). In a recent meta-analysis of sexual dysfunctions in psoriatic women, it has been found that female patients have bigger odds of dysfunctions (OR 2.67, 95% CI) and lower scores in all the domains of FSFI, except for pain (35). In one study, Molina-Leyva et al. used the Massachusetts General Hospital-Sexual Functioning Questionnaire (MGH-SFQ) which has similar domains compared to FSFI/IIEF and can be administered to both sexes. They found worse functions in all domains in patients compared to controls (10).

Female patients treated with systemic antipsoriatic agents had significantly worse lubrication, overall satisfaction, and pain associated with sexual intercourse, compared to patients treated only with topical agents. However, in men, there was no difference between patients and controls in sexual function maintenance depending on the kind of systemic antipsoriatic treatment. Apparently in men, it does not affect this area of life. As for the literature data, in a systemic review published in 2015, it was stated that the choice of treatment does not affect sexual functions (16) and in the other one that even improves them (23). We obtained opposite results, similar did Kwan et al. (36). Given the mechanism of action of certain drugs, it is highly possible that systemic antipsoriatic agents exert a negative influence on sexual activity, and some of them could be more noticeable in women. First of all, the hallmark of acitretin side effects is dryness of skin and mucosa, including vulva and vagina (37). That can certainly affect the quality of sexual intercourse. Methotrexate, another frequently prescribed drug, can probably cause erectile dysfunction due to several mechanisms: the inhibition of IL-1 and lower secretion of prolactin, causing the imbalance between estrogens and androgens, as well as inhibition of nitric oxide secretion which prevents smooth muscle cells in the vessel walls from constricting (21, 23). As for cyclosporin A, we did not find any evidence that it can cause side effects affecting sexual function. There have been reports though suggesting a beneficial influence of biological agents. In the study by Ruiz-Villaverde et al. there has been a significant improvement in FSFI and IIEF scores after 6 months of therapy (24).

However, our study showed more abnormalities in subjects undergoing systemic treatment, it should be emphasized that the topical therapy can also be troublesome due to the consistency and fragrance of the topical agents (19). We must also highlight that, unfortunately, we were not able to assess the severity or resolution of lesions after the treatment.

We did not observe any correlation between FSFI or IIEF and the age of patients, or psoriasis duration. Bardazzi et al. observed that younger women (under 46 years old) had lower FSFI scores than older women but no significant difference in FSFI between patients with or without lesions in the anogenital region (38). Wojciechowska-Zdrojowy et al. found a correlation between the age of male patients and the severity of erectile dysfunction but not with psoriasis duration (27). Interestingly, Meeuwis et al. stated that the older the patient is at the onset of genital psoriatic lesions, the worse sexual function (29).

An interesting matter is the influence of psoriasis severity on sexual life. In our study, we did not assess psoriasis activity and severity index (PASI) since it was an online survey however in other experiments the results are diversified. In the paper by Kędra et al. there was a correlation between sexual problems and PASI in both sexes (39) and in the paper by Wojciechowska-Zdrojowy et al. – in men (27). Kwan et al. assessed only body surface area (BSA) affected with lesions and found a significant difference in sexual function between patients with BSA more or less than 10 (36). Bardazzi and Nguyen et al. evaluated only female patients. The first team reported that only patients with moderate to severe psoriasis had lower FSFI than controls (38), whereas the other one found higher PASI among female patients with significantly decreased FSFI score (33, 38). In the study by Al Mazeedi and Türel et al., no significant difference was observed between patients with mild or severe psoriasis in terms of the quality of their sexual life (31, 40). On the other hand, in those who were treated successfully, and hence achieved a significant reduction in PASI, the improvement of sexual life quality was observed (12, 41).

There has always been discussion about whether the presence of psoriatic skin lesions in the anogenital area affects the patients’ sexual activity. Skin lesions in the anogenital area occur in 33–63% of women and as for men, there is less evidence, but it is reported to be about 50% (27, 42). From our personal clinical experience, we often hear that it prevents them from having satisfying intercourse however the objective data from scientific publications sometimes claim otherwise and again, are diversified. In a big analysis by Meeuwis et al. there was no significant difference in IIEF and FSFI outcomes between patients who had and did not have genital lesions (29). No correlation between erectile dysfunction and the presence of such lesions was also found by Wojciechowska-Zdrojowy or Cabete et al. In our study, almost half of the patients had lesions in the anogenital area but there was no correlation with FSFI or IIEF scores. Opposite results were reported by Kędra et al. who observed that the severity of sexual problems was associated with the lesions in the genital area, but also on the face or hands, not with the IIEF-5 (5-question version of IIEF) score (39).

We evaluated whether the questions from DLQI correspond somewhat to FSFI or IIEF. Apparently, in men, there is no correlation between IIEF scores and answers to DLQI questions. However, in females, there is a remarkable correlation between all FSFI domain scores and the total score and question no 9 from DLQI regarding sexual difficulties. That suggests DLQI could be a reliable tool in daily clinical practice for screening purposes.

Lastly, many studies mention the origin of sexual dysfunctions in psoriatics: is it due to the skin lesions themselves, or psychological impact of the dermatosis, or the associated diseases and drugs that influence sexual health? All in all, what matters in the first place is to screen the patients for such dysfunctions to distinguish a group of patients requiring help to begin the management as soon as possible. Only after the identification of subjects that report on such problems, they can be further referred to other specialists depending on the possible nature of the dysfunction. Addictions, especially alcohol abuse or heavy smoking, should be also eliminated because not only do they affect skin conditions but also sexual health (12, 25). According to the available literature, most attention should be paid to patients with depression and anxiety as they are probably most strongly related to sexual dysfunctions (22).

Apart from the sexual dysfunctions that are the main aim of this research, we gathered some data on sexual behavior in psoriatic patients. In our cohort, the significant majority of patients stayed in a relationship (married or domestic) and were heterosexual similar to the control group. The same observation was made by Armstrong et al. (43, 44). We did not observe any correlation between FSFI or IIEF and the place of patients’ residence. Nguyen on the other hand found lower FSFI scores in women from cities compared to the countryside (33). It was suggested to be related to greater exposure to psychological stress within the urban environment (33).

The main limitation of our study is that we could not assess the psoriasis severity due to the online character of our experiment. The second limitation is the relatively low number of subjects involved, yet the highest among the studies on the same topic from our country. Another point is the lack of doctor-verified information on the outcome of the introduced treatment. The interpretation of results and comparison between the groups from the previous available studies is also often difficult due to the different questionnaires used and various inclusion criteria of patients.

## 5 Conclusion

Our study has shown that in about half of psoriatic patients, the disease affects their sexual life. The majority of questioned female psoriatic patients had sexual dysfunction according to the FSFI, particularly they had worse satisfaction with their sexual life and less sexual desire compared to women without psoriasis. Moreover, female patients treated with systemic antipsoriatic agents have significantly worse lubrication, satisfaction with sexual life, and pain. As for the male psoriatic patients, the majority did not have sexual dysfunction according to IIEF, however, they had significantly worse overall satisfaction with their sexual life and confidence to keep the erection. Systemic antipsoriatic treatment does not probably influence sexual dysfunctions in men. The attention must be paid to the matter of sexual dysfunctions in psoriatic patients and they should be screened using simple tools during dermatological appointments. Our study showed that the question included in DLQI is reliable in female patients. Both psychological conditions, as well as physical comorbidities, should be evaluated as factors contributing to sexual dysfunctions.

## Data availability statement

The raw data supporting the conclusions of this article will be made available by the authors, without undue reservation.

## Ethics statement

The studies involving humans were approved by Medical University of Bialystok, Bioethical Committee. The studies were conducted in accordance with the local legislation and institutional requirements. The participants provided their written informed consent to participate in this study.

## Author contributions

JN: Conceptualization, Data curation, Investigation, Methodology, Project administration, Resources, Writing – original draft, Writing – review & editing. AK: Investigation, Methodology, Writing – review & editing. MN: Investigation, Methodology, Writing – review & editing. PP: Investigation, Writing – original draft, Writing – review & editing. AB: Supervision, Writing – review & editing. TK: Formal analysis, Software, Visualization, Writing – review & editing. ZP: Writing – review & editing. IF: Supervision, Writing – review & editing.
