# Supplementary material for: Sexual dysfunctions in psoriatic patients
Source: Front Public Health. 2024 Apr 17;12:1339196. doi: 10.3389/fpubh.2024.1339196 (PMC11061483; doi:10.3389/fpubh.2024.1339196)
Supplement: Supplementary file 1 [file Table_1.pdf]

Supplementary files

Table 1. Mean IIEF questionnaire results.

**Question**	**Controls (n=21)**	**Study Group (n=33)**
Q1	3.11±0.51	2.55±0.42
Q2	2.82±0.55	2.65±0.43
Q3	2.95±0.56	2.62±0.42
Q4	2.84±0.54	2.65±0.42
Q5	2.8±0.54	2.56±0.4
Sum: Erectile function	18.65±2.78	16.6±2.17
Q6	1.7±0.39	1.44±0.28
Q7	2.78±0.52	2.21±0.39
Q8	2.4±0.5	2.2±0.37
Sum: Intercourse satisfaction	6.85±1.32	5.87±0.99
Q9	3±0.54	2.74±0.39
Q10	3.21±0.51	2.75±0.37
Sum: Orgasmic function	6.22±1.03	5.47±0.72
Q11	3.35±0.26	3.53±0.24
Q12	3.6±0.82	3.32±0.19
Sum: Sexual desire	6.95±0.4	6.85±0.41
Q13	**3.63±0.27**	**2.82±0.23 ***
Q14	**3.65±0.27**	**2.97±0.22 ***
Overall satisfaction	**7.26±0.55**	**5.79±0.43 ***
Q15	**4.18±0.24**	**3.42±0.19 ***
TOTAL SCORE	*46.2±5.68*	*40.21±4.4 (p=0.1)*

* and **bold font** means statistically significant differences compared to the controls with p<0.05; *italics* means trend; Q means the question number; sum means the summary outcome for each domain; n means the number of subjects in each subgroup.

Patients obtained significantly lower results in question no 13,14,15 and in overall satisfaction with sexual life compared to controls. There was a downward trend in the total score for patients compared to controls.

Table 2. The results of IIEF questionnaire after the division of male patients according to the antipsoriatic treatment (topical vs systemic).

Men	Topical (n=18)	Systemic (n=15)
Q1	2.68±0.55	2.33±0.67
Q2	2.84±0.57	2.4±0.65
Q3	2.65±0.56	2.67±0.6
Q4	2.74±0.56	2.53±0.64
Q5	2.58±0.54	2.53±0.58
Sum: Erectile function	16.84±2.91	16.07±3.37
Q6	1.63±0.4	1.2±0.37
Q7	2.21±0.5	2.2±0.63
Q8	2.16±0.48	2.27±0.63
Sum: Intercourse satisfaction	6±1.33	5.7±1.52
Q9	2.95±0.5	2.47±0.63
Q10	2.84±0.48	2.5±0.59
Sum: Orgasmic function	5.79±0.98	5.1±1.22
Q11	3.37±0.32	3.74±0.36
Q12	2.98±0.22	3.82±0.3
Sum: Sexual desire	6.32±0.52	7.53±0.64
Q13	2.79±0.29	2.88±0.39
Q14	2.84±0.29	3.14±0.34
Sum: Overall satisfaction	5.63±0.56	6±0.69
Q15	3.32±0.27	3.6±0.28
TOTAL SCORE	40.58±5.85	40.27±6.89

Q means the question number; sum means the summary outcome for each domain; n means the number of subjects in each subgroup. There were no statistically significant differences between the groups in any question.

Table 3. Mean FSFI questionnaire results.

Question	Controls (n=54)	Study Group (n=47)
Q1	3.07±0.15	2.82±0.2
Q2	**2.98±0.15**	**2.51±0.16 ***
Sum: Desire	*3.62±0.17*	*3.15±0.19 (p=0.052)*
Q3	*3.17±0.27*	*2.55±0.28 (0.068)*
Q4	2.68±0.25	2.3±0.24
Q5	2.72±0.24	2.42±0.26
Q6	2.61±0.26	2.47±0.29
Sum: Arousal	3.28±0.29	2.94±0.31
Q7	3.1±0.28	3.02±0.32
Q8	3.13±0.29	3.33±0.31
Q9	2.72±0.28	2.91±0.32
Q10	3±0.29	3.11±0.34
Sum: Lubrication	3.55±0.33	3.71±0.37
Q11	2.6±0.27	2.84±0.29
Q12	2.85±0.28	2.98±0.3
Q13	2.65±0.26	2.6±0.26
Sum: Orgasm	3.24±0.31	3.38±0.32
Q14	2.98±0.29	2.8±0.29
Q15	2.74±0.26	2.74±0.26
Q16	**3.19±0.19**	**2.56±0.25 ***
Sum: Satisfaction	3.56±0.28	3.24±0.3
Q17	2.67±0.29	2.91±0.32
Q18	2.82±0.3	2.98±0.32
Q19	2.93±0.28	2.92±0.31
Sum: Pain	3.36±0.34	3.55±0.38
TOTAL SCORE	20.72±1.58	20.11±1.72

* and **bold font** means statistically significant differences compared to the controls with $p<0.05$; *italics* means trend; Q means the question number; sum means the summary outcome for each domain; n means the number of subjects in each subgroup.

Patients obtained significantly lower score in question no 2 and 16 compared to controls. There was a downward trend in the desire domain score and question no 3 for patients compared to controls.

Table 4. The mean results of FSFI questionnaire after the division of female patients according to the antipsoriatic treatment (topical vs systemic).

**Women**	**Topical (n=32)**	**Systemic (n=15)**
Q1	2.84±1.12	2.71±0.42
Q2	2.45±0.17	2.81±0.38
Sum: Desire	3.17±0.19	3.25±0.47
Q3	2.77±0.33	2.14±0.53
Q4	2.36±0.277	2.14±0.51
Q5	2.58±0.3	2.07±0.5
Q6	2.74±0.34	2.1±0.55
Sum: Arousal	3.14±0.36	2.51±0.62
Q7	**3.48±0.31**	**2.1±0.52 ***
Q8	3.64±0.37	2.64±0.55
Q9	**3.29±0.37**	**2±0.53 ***
Q10	**3.55±0.37**	**2.2±0.58 ***
Sum: Lubrication	**4.17±0.43**	**2.68±2.42 ***
Q11	3.16±0.35	2.14±0.52
Q12	3.29±0.34	2.29±0.57
Q13	2.81±0.3	2.1±0.52
Sum: Orgasm	3.7±0.37	2.63±0.63
Q14	3.07±0.35	2.21±0.53
Q15	**3.16±0.28**	**1.84±0.49 ***
Q16	**2.87±0.27**	**1.83±0.48 ***
Sum: Satisfaction	**3.64±0.34**	**2.32±0.57 ***
Q17	3.23±0.36	2.21±0.61
Q18	3.32±0.37	2.25±0.61
Q19	3.29±0.36	2.29±0.59
Sum: Pain	**4.03±0.28**	**2.53±0.52 ***
TOTAL SCORE	21.76±1.9	16.13±3.4

* and **bold font** means statistically significant differences compared to the controls with $p<0.05$; Q means the question number; sum means the summary outcome for each domain; n means the number of subjects in each subgroup.

Patients obtained significantly lower score in question no 7, 9, 10, 15, 16 and in scores for the following domains lubrication, satisfaction and pain, compared to controls.
